# Hernias of the Urinary Bladder

**Published:** 1916-06

**Authors:** Aime Paul Heineck

**Affiliations:** Professor of Surgery Chicago College of Medicine and Surgery, Surgeon to Rhodes Avenue, Jefferson Park and Frances Willard Hospitals


### Journal-Record of Medicine

Successor to Atlanta Medical and Surgical Journal, Established 1855 and Southern Medical Record, Established 1870

**OWNED BY THE ATLANTA MEDICAL JOURNAL COMPANY**

PUBLISHED MONTHLY

*Official Organ Fulton County Medical Society, State Examing Board, Atlanta, Birmingham and Atlantic Railroad Surgeon's Association, Chattahoochee Valley Medical and Surgical Association, Etc.*

RICHARD R. DALY, M. D., Editor

A. W. STIRLING, M. D., C. M., D. P. H.; J. S. HURT, B. Ph., M.D
GEO. M. NILES, M. D., W. J. LOVE, M. D., (Ala.); Associate Editors

E. W. ALLEN, Business Manager

**COLLABORATORS**

W. F. WESTMORELAND, M. D., General Surgery
F. W. McRAE, M. D., Abdominal Surgery
ALLEN H. BUNCE, Medical Societies
E. B. BLOCK, M. D., Diseases of the Nervous System
MICHAEL HOKE, M. D., Orthopedic Surgery
CYRUS W. STRICKLER, M. D., Legal Medicine and Medical Legislation
E. C. DAVIS, A. B., M. D., Obstetrics
E. G. JONES, A. B., M. D., Gynecology
ALLEN H. BUNCE, M. D., Pathology and Bacteriology
R. T. DORSEY, JR., B. S., M. D., Medicine
L. M. GAINES, A. B., M. D., Internal Medicine
GEO. C. MIZELL, M. D., Diseases of the Stomach and Intestines
L. B. CLARKE, M. D., Pediatrics
EDGAR PAULLIN, M. D., Opsonic Medicine
THEODORE TOEPEL, M. D., Mechano Therapy
R. R. DALY, M. D., Diseases of the Eye, Ear, Nose and Throat
E. G. BALLENGER, M. D., Diseases of the Genito-Urinary Organs

VOL. LXIII. ATLANTA, GA., JUNE, 1916. No. 3

### HERNIAS OF THE URINARY BLADDER.

By Aime Paul Heineck, Professor of Surgery Chicago College of Medicine and Surgery, Surgeon to Rhodes Avenue, Jefferson Park and Frances Willard Hospitals.

The permanent or temporary escape of a part or the whole of the urinary bladder, through any of the usual or unusual hernial orifices, is uncommon. Nevertheless, many cases have been published and a much larger number have been allowed to pass without record. In a long series of hernia operations, every surgeon is certain to meet with some instances of hernia of the bladder. The urinary bladder in part or in its entirety is present in 1 per cent. of all hernias.

Though the term hernia implies the presence of a hernial opening, of a hernial sac, sac-contents and sac-coverings, we know that in many hernias of the urinary bladder, the sac is either incomplete or totally absent.

To designate the clinical entity under consideration, we fail to find any other term more appropriate, more sanctioned by long usage than that of hernia of the urinary bladder.

Many operators have unknowingly punctured, incised, ligated or removed a herniated bladder-process and then closed the hernial canal and operative wound in the usual way. Bladder protrusions have been excised by mistake for hernial sacs, or stitches used to close hernial canals have been passed too deeply and found at the necropsy to have caught the bladder.

An analysis of all the vesical hernias reported with sufficient data in the English, French and German languages from 1896 to 1914, inclusive (literature to which access can be had at the John Crerar Library, Chicago, Ill.) and also some unpublished personal cases (in all one hundred and fifty-nine patients, representing one hundred and sixty-four vesical hernias), justifies, in our opinion, the following conclusions:

1. The urinary bladder, in part or in its entirety, may escape from the abdominal and abdomino-pelvic cavities through any of the uncommon or common hernial orifices of the lower abdominal wall.

2. Hernias of the urinary bladder occur in both sexes, at all ages, and in all races. They are congenital or acquired, recurrent, recent or of some standing; almost always unilateral, very rarely bilateral. Like other hernias, they vary in shape, size, rate of growth and in the discomfort and disability which they entail.

3. In the female, vesical hernias occur in nulliparae, primiparae and multiparae; they occur previous to, during, or after gestation and between gestations. They neither interfere with gestation nor disturb parturition.

4. According to anatomical site, vesical hernias are designated as hernias of the linea alba, of the obturator, femoral or inguinal regions. Anatomical relations justify the further subdividing of the two latter types into interstitial or intra-parietal, direct or indirect, complete or incomplete, pudendal or scrotal.

5. The relation of the herniated bladder-process to the serous membrane lining the peritoneal cavity is well expressed by the terms: Intra-peritoneal, para-peritoneal and extra-peritoneal. These designations are serviceable from the viewpoint of etiology, symptomatology and treatment.

6. According to clinical manifestations, hernias of the urinary bladder are reducible, irreducible, in__amed or strangulated.

7. A vesical hernia may be single, double, or one of two or more hernias located on the same or opposite sides of the body, having dissimilar contents, and presenting like or unlike anatomical and clinical characteristics. Thus, the same patient may present an inguinal cystocele and a femoral epiplocele, a reducible femoral vesical hernia and an irreducible inguinal intestinal hernia. Case reports of an inguinal vesical hernia on one side co-existing with an inguinal enterocele, epiplocele or entero-epiplocele on opposite side of the body are not uncommon.

8. As etiological factors, in the causation of vesical hernias, the following are foremost:

a. All conditions that tend to increase intra-abdominal pressure.

b. All conditions, congenital or acquired, that weaken the abdominal wall.

c. All diseases of the lower urinary organs that impair the expulsive force of the bladder or abnormally hinder the outflow of urine.

d. Pre-existing hernias and hernial sacs of pre or post-natal origin.

9. The pre-operative signs and symptoms may be unmistakable, vague or absolutely wanting. In addition to such symptoms as are common to all other hernias, vesical hernias present peculiar suggestive and positive manifestations of their existence. Chief among the former are such disturbances of micturition as the following: Frequent, painful and difficult urination, vesical tenesmus, urgent desire to urinate caused by pressure upon hernial swelling and two-step urination. Chief among the positive manifestations are: A hernial swelling increasing in size with urinary retention and decreasing with urination; increasing in size with air or water-distention of the bladder and decreasing upon withdrawal of these agents; passage of a sound into the herniated bladder-process by way of urethra and bladder; cystoscopic demonstration of the vesical orifice of the herniated bladder-process.

10. The herniated bladder-process may be the sole content of the hernial swelling, or merely one of the associated contents. In addition to a bladder-process, a hernial swelling may contain, a part of one or more of the following organs: Ureter, Fallapian tube, ovary, appendix, vermiformis or appendix epiploicae, omentum, and small or large intestine.

11. The herniated bladder-process may be free or adherent to surrounding tissues or organs, structurally normal or present degenerative, inflammatory or neoplastic changes; may be the seat of atrophy, hypertrophy, catarrh, gangrene, tuberculosis or carcinoma. and may or may not communicate freely with the general vesical cavity. The herniated process of bladder may contain one or more calculi.

12. The vesical hernia may be the sole existing anomaly, or it may be one of two or more, congenial or acquired, pathological states, having or not having any relationship of cause or effect to the hernia (cryptorchism, vaginal cystocele, prolapsus uteri, prostatic hypertrophy, etc.).

13. Truss treatment for hernias of the bladder is not curative, is often productive of discomfort and may injuriously affect the structure of the bladder-wall.

14. In patients over ten years of age, all hernias, irrespective of anatomical site, clinical condition or contents, should, in the

21. In inguinal and femoral hernia operations, after careful opening and isolation of the sac, see that the latter consists preferably of peritoneum only, and that its neck be freed from all other structures. Neck of sac should not be twisted, as by so absence of a constitutional state contra-indicating operations of election, be subjected to an operation for radical cure.

15. Clinical conditions so closely simulating hernias of the urinary bladder that a positive diagnosis without operation appears impossible, should be subjected to operative treatment. Only benefit can be derived from adherence to this rule. A diagnosis is established and a cure is effected.

16. All hernias of the urinary bladder, irrespective of sex, age or social condition of patient, irrespective of size, shape anatomical site or clinical type, call for operative treatment. Operative treatment is free from danger and is curative. The only contra-indications to operative treatment are: Extreme old age and the co-existence of a pathological state or states contra-indicating operation of election. Operative treatment is the only rational treatment of hernia in the adult.

17. Operative intervention is indicated in all incarcerated and in all strangulated hernias of the bladder.

18. In all hernias, the ideal time for operation is previous to the development of degenerative or other pathological changes in the herniated organ or organs and previous to the occurrence of any of the various complications incident to hernias.

19. Women who suffer from any form of hernia should be carefully watched before, during and after their confinement, so as to prevent or rather minimize any undue strain upon weak regions of the abdominal wall. Those women, at the close of lactation or towards the end of the first year following their confinement, should, in the absence of contra-indications, be subjected to an operation for radical cure of the hernia. In the female, the inguinal rings are comparatively small. They can without inconvenience to the patient be closed.

20. The most popular and efficient modern hernia operations permit a full view of operative field and allow such a careful examination of hernial rings, canals, and surrounding structures, that a prolapsed or herniated bladder-process rarely escapes detection.

doing the bladder is drawn towards the hernial opening and is liable to be included in the ligature. Necrosis and peritonitis result therefrom.

22. In the course of a hernia operation, if, after opening the sac and reduction of its contents, there appears a second sac, it is not to be opened, unless the introduction of a sound in the bladder shows the complete independence of this sac from the urinary reservoir.

23. In hernias of the urinary bladder, first expose and free the herniated organ or organs, and then reduce it into the abdomino-pelvic cavity. Follow this by suppressing the hernial sac if one be present, and then strengthen, according to an approved method, the weakened hernial area. Resection of the herniated bladder-process is only exceptionally indicated. When performed, it calls for immediate reconstitution of the urinary reservoir.

24. During hernia operations, the wounding of the urinary bladder can, to a large extent, be prevented by careful operating and by keeping in mind that this clinical entity occurs.

25. Wounds of the urinary bladder inflicted during the course of hernia operations, give a good prognosis if they be immediately repaired and if the post-operative treatment be instituted appropriately. In the repair of bladder wounds, two or three layers of continuous or interrupted absorbable sutures give satisfactory results. Bladder suturing is to be followed by refection of the abdominal wall of the hernial area.

26. If within twenty-four to forty-eight hours after a hernia operation on a healthy subject the catheterized urine contains blood, determine the origin of the blood. If a bladder-injury be present, open the hernial operative wound or laparotomize or do both and repair the injury.

27. The mortality of operations for the radical cure of hernia, if performed at an opportune time by a rapid and skillful operator competently assisted, is practically nil. Coley operated 1000 consecutive cases of hernia without a single death.

28. The operative treatment of hernias of the urinary bladder is highly satisfactory.

